# 1334. Outcomes Among Influenza and SARS-CoV-2 Infection in Hospitalized Adults Age ≥ 50 Years and with Underlying Chronic Obstructive Pulmonary Disease (COPD) or Congestive Heart Failure (CHF)

**DOI:** 10.1093/ofid/ofab466.1526

**Published:** 2021-12-04

**Authors:** Megan Taylor, Ashley Tippett, Laila Hussaini, Luis Salazar, Caroline Ciric, Olivia Reese, Laurel Bristow, Vikash Patel, Wensheng Li, Hui-mien Hsiao, Kathy Stephens, Theda Gibson, Ariel Kay, Andrew Cheng, David L Swerdlow, Robin Hubler, Ben Lopman, Christina A Rostad, Larry Anderson, Nadine Rouphael, Nadine Rouphael, Evan J Anderson

**Affiliations:** 1 Emory University School of Medicine, Atlanta, Georgia; 2 Pfizer, Inc, New York, NY; 3 Pfizer Inc, Collegeville, PA; 4 Rollins School of Public Health, Emory University, Atlanta, GA; 5 Emory University, Atlanta, GA; 6 Emory University, Atlanta VA Medical Center, Atlanta, Georgia

## Abstract

**Background:**

A significant burden of disease exists for adults infected with influenza (flu) and SARS-CoV-2, which causes COVID-19. However, data are limited comparing outcomes between hospitalized adults infected with these viruses.

**Methods:**

Over the course of 3 consecutive winter respiratory viral seasons, adults ≥ 50 years of age admitted with acute respiratory tract infections (ARI) and adults of any age with COPD or CHF-related admissions were enrolled from 2 Atlanta area hospitals. For the 2018-19 and 2019-20 seasons, participants were approached in the hospital. If the participant enrolled, nasopharyngeal (NP) and oropharyngeal (OP) swabs were collected and tested using BioFire® FilmArray® respiratory panel. Due to the COVID-19 pandemic in 2020-21 and limitations involving participant contact, only NP standard of care (SOC) swabs were collected. A comprehensive medical chart review was completed for each subject which encompassed data on their hospitalization, past medical history, and vaccination history. Co-infected patients were excluded from the analyses.

**Results:**

Of the eligible participants, 118 were flu positive (three RSV-influenza co-infections were excluded) and 527 were COVID-19 positive. Median age was lower for the flu cohort at 62 (IQR 56-71) than those with COVID-19 (67, IQR 59-77) (p < 0.0001). Length of stay (LOS) was shorter in flu-infected patients (median 3 d, IQR 2-6), but was longer for COVID-19 patients (median 5 d, IQR 3-10). ICU admission occurred in 20% of those with flu, and among those admitted to the ICU mechanical ventilation (MV) occurred in 12.5%. ICU admission and MV was significantly higher for those with COVID-19, with 28% of patients admitted to the ICU and 47% of those requiring MV. Among patients with COVID-19, 8.9% died. This was significantly higher than that of flu (3.4%) (p=0.008). Hospital discharge occurred more frequently to a nursing home or LTCF with COVID-19 (10.3%) than with flu (0%) (p< 0.0001).

Table 1. Breakdown of age, hospitalization course, and discharge disposition for participants diagnosed with influenza or COVID-19 during hospitalization.

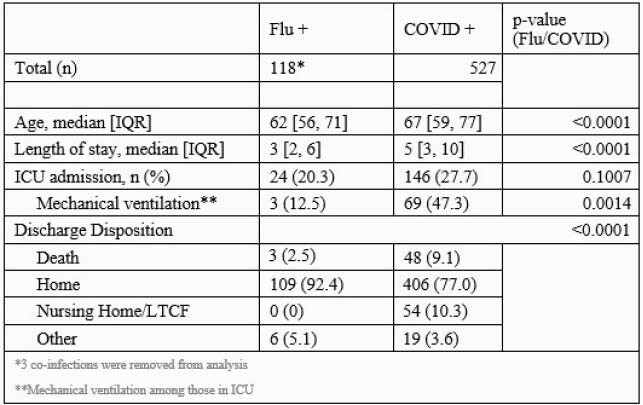

**Conclusion:**

COVID-19 resulted in a longer hospital admission, a greater chance of ICU admission and MV as compared to flu. Additionally, COVID-19 participants had a high rate of discharge to a nursing home/LTCF and a significantly higher risk of death. While the clinical course was not as severe as COVID-19, influenza contributed a significant burden.

**Disclosures:**

**David L. Swerdlow, MD**, **Pfizer Vaccines** (Employee) **Robin Hubler, MS**, **Pfizer Inc.** (Employee) **Christina A. Rostad, MD**, **BioFire Inc, GSK, MedImmune, Micron, Janssen, Merck, Moderna, Novavax, PaxVax, Pfizer, Regeneron, Sanofi-Pasteur.** (Grant/Research Support, Scientific Research Study Investigator, Research Grant or Support)**Meissa Vaccines** (Other Financial or Material Support, Co-inventor of patented RSV vaccine technology unrelated to this manuscript, which has been licensed to Meissa Vaccines, Inc.) **Larry Anderson, MD**, **ADVI** (Consultant)**Bavarian Nordic** (Consultant)**Novavax** (Consultant)**Phizer** (Grant/Research Support, Scientific Research Study Investigator)**Sciogen** (Research Grant or Support) **Nadine Rouphael, MD**, **pfizer, sanofi, lily, quidel, merck** (Grant/Research Support) **Nadine Rouphael, MD**, Lilly (Individual(s) Involved: Self): Emory Study PI, Grant/Research Support; Merck (Individual(s) Involved: Self): Emory study PI, Grant/Research Support; Pfizer: I conduct as co-PI the RSV PFIZER study at Emory, Research Grant; Pfizer (Individual(s) Involved: Self): Grant/Research Support, I conduct as co-PI the RSV PFIZER study at Emory; Quidel (Individual(s) Involved: Self): Emory Study PI, Grant/Research Support; Sanofi Pasteur (Individual(s) Involved: Self): Chair phase 3 COVID vaccine, Grant/Research Support **Evan J. Anderson, MD**, **GSK** (Scientific Research Study Investigator)**Janssen** (Consultant, Scientific Research Study Investigator, Advisor or Review Panel member)**Kentucky Bioprocessing, Inc** (Advisor or Review Panel member)**MedImmune** (Scientific Research Study Investigator)**Medscape** (Consultant)**Merck** (Scientific Research Study Investigator)**Micron** (Scientific Research Study Investigator)**PaxVax** (Scientific Research Study Investigator)**Pfizer** (Consultant, Grant/Research Support, Scientific Research Study Investigator)**Regeneron** (Scientific Research Study Investigator)**Sanofi Pasteur** (Consultant, Scientific Research Study Investigator)

